# Extracellular ATP and the P2X_7 _receptor in astrocyte-mediated motor neuron death: implications for amyotrophic lateral sclerosis

**DOI:** 10.1186/1742-2094-7-33

**Published:** 2010-06-09

**Authors:** Mandi Gandelman, Hugo Peluffo, Joseph S Beckman, Patricia Cassina, Luis Barbeito

**Affiliations:** 1 Neurodegeneration Laboratory, Institut Pasteur, Montevideo, Uruguay; 2 Departamento de Histología, Facultad de Medicina, Universidad de la República, Montevideo, Uruguay; 3 Linus Pauling Institute, Oregon State University, Corvallis, Oregon 97331, USA; 4 Instituto de Investigaciones Biológicas Clemente Estable, Montevideo, Uruguay

## Abstract

**Background:**

During pathology of the nervous system, increased extracellular ATP acts both as a cytotoxic factor and pro-inflammatory mediator through P2X_7 _receptors. In animal models of amyotrophic lateral sclerosis (ALS), astrocytes expressing superoxide dismutase 1 (SOD1^G93A^) mutations display a neuroinflammatory phenotype and contribute to disease progression and motor neuron death. Here we studied the role of extracellular ATP acting through P2X_7 _receptors as an initiator of a neurotoxic phenotype that leads to astrocyte-mediated motor neuron death in non-transgenic and SOD1^G93A ^astrocytes.

**Methods:**

We evaluated motor neuron survival after co-culture with SOD1^G93A ^or non-transgenic astrocytes pretreated with agents known to modulate ATP release or P2X_7 _receptor. We also characterized astrocyte proliferation and extracellular ATP degradation.

**Results:**

Repeated stimulation by ATP or the P2X_7_-selective agonist BzATP caused astrocytes to become neurotoxic, inducing death of motor neurons. Involvement of P2X_7 _receptor was further confirmed by Brilliant blue G inhibition of ATP and BzATP effects. In SOD1^G93A ^astrocyte cultures, pharmacological inhibition of P2X_7 _receptor or increased extracellular ATP degradation with the enzyme apyrase was sufficient to completely abolish their toxicity towards motor neurons. SOD1^G93A ^astrocytes also displayed increased ATP-dependent proliferation and a basal increase in extracellular ATP degradation.

**Conclusions:**

Here we found that P2X_7 _receptor activation in spinal cord astrocytes initiated a neurotoxic phenotype that leads to motor neuron death. Remarkably, the neurotoxic phenotype of SOD1^G93A ^astrocytes depended upon basal activation the P2X_7 _receptor. Thus, pharmacological inhibition of P2X_7 _receptor might reduce neuroinflammation in ALS through astrocytes.

## Background

Amyotrophic lateral sclerosis (ALS) is characterized by the progressive degeneration of motor neurons in the spinal cord, brainstem and motor cortex, leading to respiratory failure and death of affected patients within a few years of diagnosis [1]. The discovery of mutations in the gene encoding the antioxidant enzyme Cu/Zn superoxide dismutase-1 (SOD1) in a subset of patients with familial ALS has led to the development of transgenic animal models expressing different SOD1 mutations [2]. These animal models recapitulate the human disease, exhibiting aberrant oxidative chemistry [3,4], neuroinflammation [5], endoplasmic reticulum stress [6], glutamate excitotoxicity [7], mitochondrial dysfunction [8] and protein misfolding and aggregation [9]. However, the mechanisms behind motor neuron death are unknown.

Accumulating evidence indicates that non-neuronal cells contribute to motor neuron dysfunction and death in ALS by the maintenance of a chronic inflammatory response [10-12]. Activated microglia accumulate in the spinal cord, producing inflammatory mediators and reactive oxygen and nitrogen species [11]. Astrocytes, the most abundant cells in the adult nervous system, also become reactive and display inflammatory features [12,13]. Remarkably, astrocytes carrying SOD1 mutations release soluble factors that selectively induce the death of motor neurons [14-18]. Astrocytes carrying the SOD1^G93A ^mutation display mitochondrial dysfunction, increased nitric oxide and superoxide production and altered cytokine liberation profile [14,17,19-22]. Thus, SOD1 mutation causes astrocytes to display a neurotoxic phenotype dependent on autocrine/paracrine pro-inflammatory signaling and increased oxidative and nitrative stress [14,19,23].

In the central nervous system, extracellular adenosine-5'-triphosphate (ATP) has physiological roles in neurotransmission, glial communication, neurite outgrowth and proliferation [24]. Extracellular ATP levels markedly increase in the nervous system in response to ischemia, trauma and inflammatory insults [25-28]. In these cases, ATP is a potent immunomodulator regulating the activation, migration, phagocytosis and release of pro-inflammatory factors in immune and glial cells.

Extracellular ATP effects are mediated by metabotropic (P2Y) and ionotropic (P2X) receptors, both widely expressed in the nervous system [24]. The P2X_7 _receptor (P2X_7_r) is a ligand-gated cation channel that elicits a robust increase in intracellular calcium [29]. Of all P2 receptors, P2X_7_r has the highest EC_50 _(>100 μM) for ATP. The high extracellular concentrations of ATP needed to activate P2X_7_r are most likely to arise under pathological conditions. In the normal rodent brain, P2X_7_r expression in astrocytes is generally low, but quickly upregulated in response to brain injury or pro-inflammatory stimulation in cell culture conditions [30-32]. In astrocytes, P2X_7_r activation can potentiate pro-inflammatory signaling, as it enhances IL-1β-induced activation of NF-κB and AP-1, leading to increased production of nitric oxide as well as increased production of the chemokines MCP-1 and IL-8 [33,34].

Inhibition of P2X_7_r and other P2X receptors is neuroprotective in animal models of experimental autoimmune encephalomyelitis and Alzheimer's and Huntington's disease [35-37]. In addition, P2X_7_r mediates motor neuron death after traumatic spinal cord injury, and systemic inhibition in vivo protects motor neurons and promotes functional recovery [25,38]. In ALS patients as well as SOD1^G93A ^animals, increased immunoreactivity for P2X_7_r has been found in spinal cord microglia [39,40]. Furthermore, SOD1^G93A ^microglia in culture display an increased sensitivity to ATP, and P2X_7_r activation drives a pro-inflammatory activation that leads to decreased survival of neuronal cell lines [41].

Despite the recognized detrimental role of extracellular ATP and P2X_7_r signaling during nervous system pathology, little is known about its effects on astrocytes or its possible role in ALS. We investigated whether ATP acting through P2X_7_r could trigger a neurotoxic transformation of astrocytes leading to motor neuron death. We also explored whether ATP signaling in SOD1^G93A ^astrocytes is involved in the maintenance of their neurotoxic phenotype towards motor neurons.

## Methods

### Chemicals and reagents

Cell culture media and reagents, 5-bromo-2-deoxyuridine (BrdU), primary antibody against BrdU and secondary antibodies were purchased from Invitrogen (Life Technologies). The Malachite Green Phosphate Assay kit was purchased from Cayman Chemical. All other reagents were from Sigma.

### Animals

Procedures using laboratory animals were in accordance with the international guidelines for the use of live animals and were approved by the Institutional Animal Care Organization of the School of Medicine, Universidad de la República (Uruguay) and by the Oregon State University IACUC.

### Primary astrocyte cultures

Astrocytes were prepared from spinal cords of 1 day old rat pups as previously described [42]. Astrocytes were plated at a density of 2 × 10^4 ^cells/cm^2 ^and maintained in Dulbecco's modified Eagle's medium supplemented with 10% fetal bovine serum, HEPES (3.6 g/L), penicillin (100 IU/mL) and streptomycin (100 μg/mL). Monolayers were >98% pure as determined by GFAP immunoreactivity and devoid of OX_42_-positive microglial cells. Transgenic SOD1^G93A ^and non-transgenic astrocytes were prepared in parallel using littermate pups previously genotyped by PCR.

### Primary motor neuron cultures

Motor neurons were prepared from embryonic day 15 rat spinal cords as previously described [42,43]. Briefly, the dorsal horns of spinal cords were dissected and incubated in 0.05% trypsin for 15 minutes at 37°C, followed by mechanical dissociation. Motor neurons were then purified by centrifugation on an Optiprep cushion, followed by isolation of p75^NTR ^expressing motor neurons by immunoaffinity selection with the IgG 192 monoclonal antibody. For co-culture experiments, astrocyte monolayers were washed twice with phosphate buffered saline (PBS) after experimental treatments and then non-transgenic motor neurons were plated on top at a density of 350 cells/cm^2^. Co-cultures were maintained for 48 hours in L15 medium supplemented with 0.63 mg/ml sodium bicarbonate, 5 μg/ml insulin, 0.1 mg/ml conalbumin, 0.1 mM putrescine, 30 nM sodium selenite, 20 nM progesterone, 20 mM glucose, 100 IU/ml penicillin, 100 μg/ml streptomycin, and 2% horse serum [42,43]. Pure motor neuron cultures were cultured for 48 hours on a polyornithine-laminin substrate in Neurobasal media supplemented with 2% horse serum, 25 μM L-glutamate, 25 μM 2-mercaptoethanol, 500 μM L-glutamine, and 2% B-27 supplement [42,43]. Survival was maintained by the addition of GDNF (1 ng/ml).

### Astrocyte treatments

**Figure 1:**
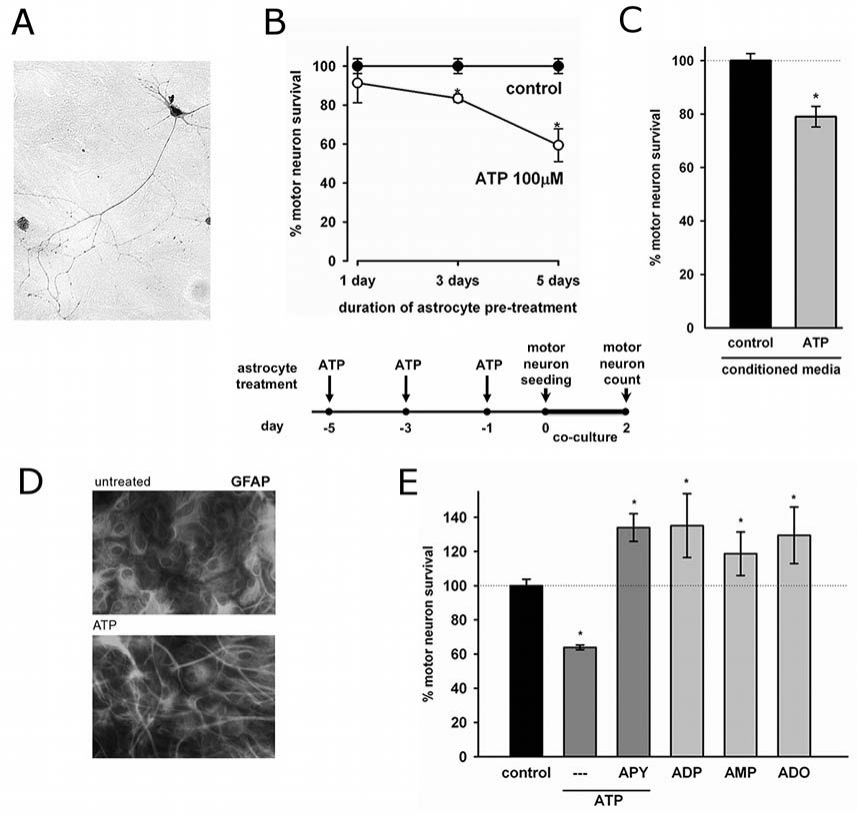
**ATP induced a neurotoxic phenotype in non-transgenic astrocytes**. (A) Motor neuron stained for p75^NTR ^cultured on the top of an astrocyte monolayer (B) Motor neuron survival in coculture with astrocytes pretreated with ATP (100 μM, top graph) as described in the diagram (bottom). Astrocytes treated for 5, 3 or 1 day(s) received 3, 2 or 1 ATP addition(s) correspondingly. (C) Survival of motor neurons in pure cultures exposed to conditioned media from control or ATP-pretreated astrocytes (100 μM, 5 days, 3 additions). (D) GFAP immunofluorescence of control and ATP-treated astrocytes (100 μM, 5 days, 3 additions) (E) Motor neuron survival in co-culture with astrocytes pretreated with ATP and apyrase (5 U/ml), ADP, AMP or Adenosine (ADO, 0.1 μM, 5 days) on motor neuron survival. Data are expressed as percentage of control, mean ± SEM from at least three independent experiments. *p < 0.05, significantly different from untreated control.

All astrocyte treatments were performed in DMEM 2% FBS for 48 hours unless otherwise stated. Stock solutions were prepared as 100× and added directly to the well after media change. Inhibitors were added 1 hour prior to subsequent treatment. As noted in Figure 1A, to determine the time-dependency of ATP exposure, media was replenished every 48 hours and 100 μM ATP was added. Thus, astrocytes treated for one day received a single ATP addition while astrocytes treated for three and five days correspondingly received 2 and 3 ATP additions.

### Production of conditioned media and treatment of pure motor neuron cultures

To produce conditioned media, astrocytes were treated with ATP 3 times during the course of 5 days. Twenty-four hours after the last treatment, monolayers were washed 3 times with PBS and then incubated for 48 hours with Neurobasal media supplemented with 2% horse serum. Conditioned media was centrifuged to remove debris, aliquoted and stored at -80°C until use. Pure motor neuron cultures were exposed to astrocyte conditioned media 3 hours after plating by replacing 50% of their complete media with conditioned media. GDNF was then added to a final concentration of 1 ng/ml.

### Motor neuron survival assessment

Motor neuron survival was assessed after 48 hours by counting all cells displaying intact neurites longer than 4 cells in diameter [42]. Counts were performed over an area of 0.9 cm^2 ^in 24-well plates. In pure cultures, motor neurons were counted under phase contrast. In co-cultures cells were fixed, immunostained for p75^NTR ^(Figure 1A) and counted [42]. In primary motor neuron cultures, the range of motor neuron death is generally limited to a subpopulation of 40 to 50% [44].

### GFAP immunofluorescence

Astrocytes grown on coverslips were fixed with ice-cold 4% paraformaldehyde in PBS for 15 minutes. Cultures were permeabilized with 0.1% Triton X-100 in PBS for 15 min and blocked for 1 hour with 10% goat serum, 2% bovine serum albumin, and 0.1% Triton X-100 in PBS. Anti-GFAP monoclonal antibody diluted in blocking solution (1:400) was incubated overnight at 4°C. After washing, cultures were incubated for 1 hour at room temperature with Alexa Fluor 488-conjugated goat anti-mouse antibody (1:500). Nuclei were stained with DAPI (1 μg/mL).

### Assessment of astrocyte proliferation

Confluent astrocyte monolayers were treated with apyrase for 48 hours in DMEM 2% FBS. At the end of the first 24 hours, BrdU (10 μg/mL) was added. BrdU immunofluorescence was performed as described for GFAP with the addition of a DNA denaturalization step with 1 M hydrochloric acid (30 min at room temperature) after permeabilization. Percentage of BrdU nuclei was calculated as the number of coincident BrdU and DAPI stained nuclei over the total number of DAPI-stained nuclei.

### Determination of ATP degradation by phosphate measurement

To determine extracellular ATP hydrolysis, extracellular phosphate production was measured with the Malachite Green Phosphate Assay kit. After the treatment, astrocyte cultures in 24 well plates were washed 3 times with a phosphate free buffer (2 mM CaCl_2_, 120 mM NaCl, 5 mM KCl, 10 mM glucose, 20 mM Hepes, pH 7.4) and incubated in 500 μl with 3 mM ATP as described [45]. After 10 minutes, an aliquot of each well was removed and phosphate was immediately measured following the manufacturer's instructions.

### Statistics

Each experiment was repeated at least three times and data are reported as mean ± SEM. Statistical analysis was performed by one-way analysis of variance, followed by a Student-Newman-Keuls test. Differences were declared statistically significant if p < 0.05. Statistics were performed using SigmaStat (Jandel Scientific, San Rafael, CA, USA).

## Results

### ATP caused non-transgenic astrocytes to induce motor neuron death

Exposure to extracellular ATP caused a neurotoxic activation of spinal cord astrocytes, which lead to death of co-cultured motor neurons in a time dependent-manner. Because ATP is quickly hydrolyzed in the extracellular media and to mimic pathological conditions with persistent ATP stimuli, we treated astrocytes repeatedly as shown in diagram in Figure 1B. Before plating motor neurons on top, astrocyte monolayers were thoroughly washed to remove any traces of the treatment. After 2 days of co-culture, motor neuron survival was assessed. Astrocytes exposed to a single addition of ATP 24 hours before co-culture showed no significant toxicity to motor neurons (Figure 1B). However, astrocytes exposed to two additions of ATP (3 and 1 days before co-culture) decreased motor neuron survival by 27 ± 17%, and astrocytes treated with three ATP additions (5, 3 and 1 days before co-culture) decreased motor neuron survival by 36 ± 1.4% (Figure 1B). In addition, conditioned media from these astrocytes applied to purified motor neuron cultures plated on a laminin substrate induced a 20% decrease in survival (Figure 1C), suggesting ATP leads to the release of a diffusible factor from astrocytes able to induce motor neuron death. Immunocytochemical analysis of these astrocytes evidenced morphological changes associated with activation, displaying long and thin processes with intense GFAP immunoreactivity as compared to the typical polygonal shape of resting astrocytes (Figure 1D).

To confirm that the effects seen on astrocytes were caused by ATP and not its degradation products ADP, AMP or adenosine (ADO), we treated astrocytes with ATP in combination with the enzyme apyrase (5 U/mL), which rapidly degrades ATP to AMP and phosphate. In this condition, the death of co-cultured motor neurons was completely prevented. Moreover, motor neuron survival increased above controls to 134 ± 8% (Figure 1E). Pretreatment of astrocytes directly with the products of ATP degradation ADP, AMP or adenosine (ADO) (0.1 μM added 3 times over five days as was done with ATP) caused an equivalent increase in astrocytic trophic support to motor neurons (Figure 1E).

### P2X_7_r activation causes astrocytes to promote motor neuron death

**Figure 2:**
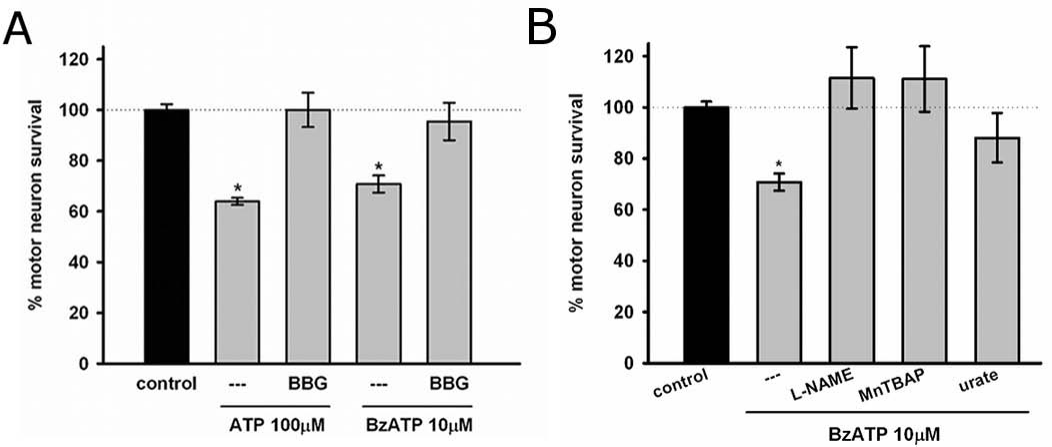
**P2X_7_r activation triggered astrocyte-mediated neurotoxicity by inducing oxidative stress**. (A) Motor neuron survival in co-culture with astrocytes pre-treated with ATP (100 μM, 5 days) or BzATP (10 μM, 48 hours) and the P2X_7_r inhibitor BBG (1 μM). (B) Motor neuron survival in co-culture with astrocytes pre-treated with NAME (1 mM), MnTBAP (0.1 mM) or urate (0.2 mM) and BzATP before co-culture. Data are expressed as the percentage of control, mean ± SEM from at least three independent experiments. *p < 0.05, significantly different from untreated control.

To investigate the role of P2X_7_r as an initiator of astrocyte-mediated motor neuron death, we used the preferential P2X_7_r agonist 2',3'-O-(4-benzoylbenzoyl)ATP (BzATP). Figure 2A shows that a 48-hour treatment of astrocytes with BzATP (10 μM) resulted in death of 30 ± 3% of co-cultured motor neurons. The effects of ATP and BzATP were prevented by the P2X_7_r antagonist BBG (1 μM), suggesting that P2X_7_r activation was required to induce the astrocyte neurotoxic phenotype (Figure 2A).

We then investigated whether the P2X_7_r-induced phenotypic change in astrocytes could be prevented by agents known to modulate oxidative and nitrative stress. BzATP-treated astrocytes were no longer toxic to motor neurons when the astrocytes were treated with the nitric oxide synthase inhibitor L-NAME (nitro-L-arginine methyl ester, 1 mM), the superoxide scavenger MnTBAP (Manganese (III) tetrakis (4-benzoic acid) porphyrin, 100 μM) and urate (200 μM) (Figure 2B). Urate efficiently scavenges peroxynitrite-derived free radicals and thereby inhibits tyrosine nitration of proteins [46,47].

### Inhibition of ATP signaling in SOD1^G93A ^astrocytes prevents astrocyte-mediated motor neuron death and cell proliferation

**Figure 3:**
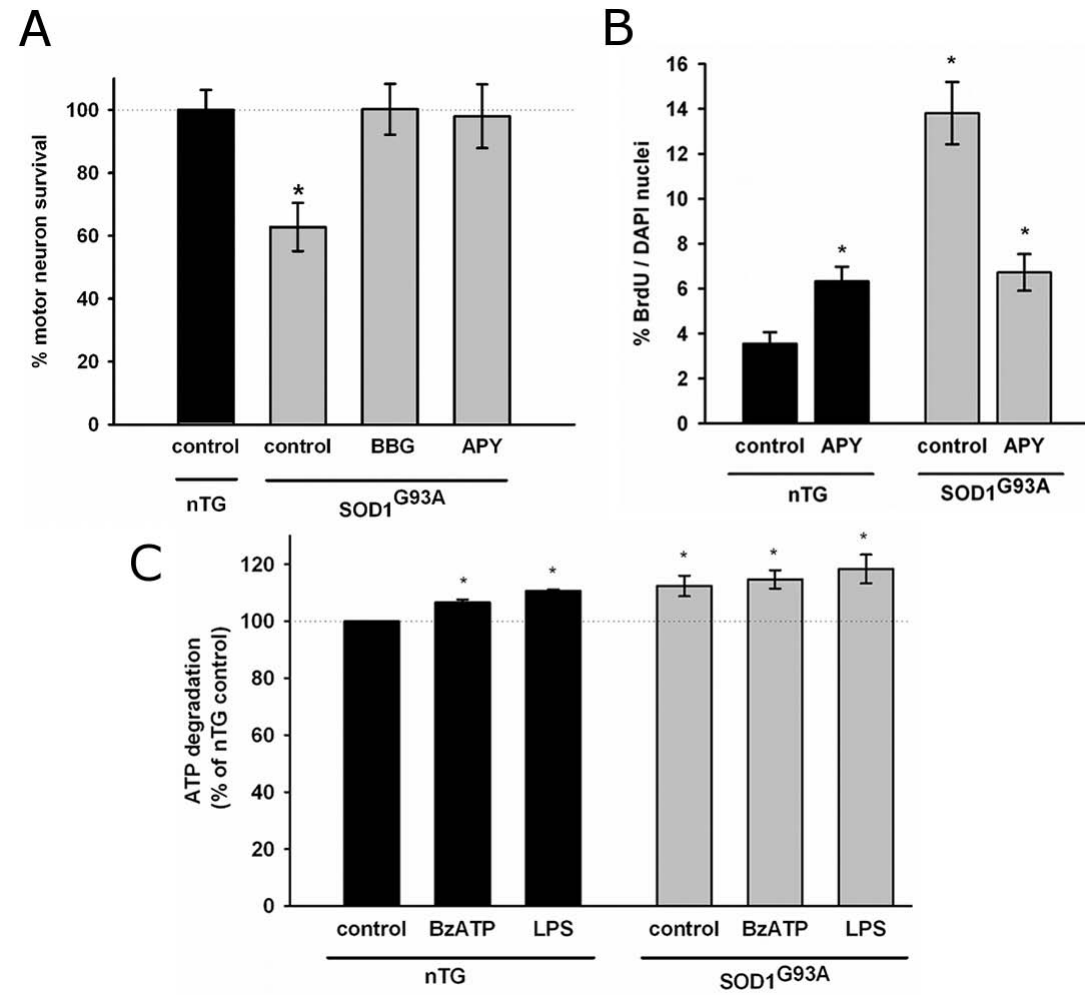
**SOD1^G93A ^astrocytes exhibit ATP-dependent neurotoxicity, proliferation, and increased ATP degradation**. (A) Motor neuron survival in co-culture with SOD1^G93A ^astrocytes pre-treated for 48 hours with the P2X_7_r inhibitor BBG (1 μM) or the ATP-hydrolyzing enzyme apyrase (5 U/ml) (B) Effect of apyrase treatment on SOD1^G93A ^astrocyte proliferation in culture. (C) Degradation of exogenously added ATP by SOD1^G93A ^or non-transgenic astrocytes astrocytes. Data are expressed as percentage of non-transgenic control, mean ± SEM from at least three independent experiments. Data are expressed as percentage of non-transgenic control, mean ± SEM from at least three independent experiments. *p < 0.05, significantly different from non-transgenic control.

Consistent with previous reports [14], spinal cord astrocytes from SOD1^G93A ^rats induced death of 37 ± 8% of co-cultured motor neurons. Remarkably, pre-incubation of SOD1^G93A ^astrocytes with apyrase to degrade endogenous extracellular ATP for 48 hours before co-culture completely prevented motor neuron death (Figure 3A). Pretreatment with the P2X_7_r inhibitor BBG also restored motor neuron survival to non-transgenic levels (Figure 3A). This suggests that P2X_7_r could be basally activated in SOD1^G93A ^astrocytes in an autocrine/paracrine manner, resulting in neurotoxicity to motor neurons.

Because purinergic signaling plays a key role in modulating astrocyte proliferation in pathological conditions [48,49], we assessed whether increased ATP signaling was involved in the proliferation of SOD1^G93A ^astrocytes. Cultured SOD1^G93A ^astrocytes showed a 4- to 5-fold increased proliferation rate as compared with non-transgenic astrocytes (Figure 3B). Proliferation in SOD1^G93A ^astrocytes was decreased in half by apyrase to the same level as apyrase-treated non-transgenic astrocytes (Figure 3B). The small increase in proliferation of non-transgenic astrocytes caused by apyrase could be caused by generation of adenosine, which has been shown to stimulate proliferation of astrocytes [49,50].

The increase in ATP signaling observed in SOD1^G93A ^astrocytes did not result from decreased extracellular degradation. On the contrary, ATP hydrolysis was 11% greater in SOD1^G93A ^astrocytes (Figure 3C). Similarly, stimulation with LPS or BzATP induced a comparable increase in ATP degradation in non-transgenic astrocytes. In SOD1^G93A ^astrocytes, these agents did not induce further ATP degradation.

## Discussion

Extracellular ATP has become increasingly recognized to have a major role in neurodegenerative processes, but its role in astrocyte-mediated neuronal death has not been explored. Here, we found that spinal cord astrocytes assume a neurotoxic phenotype in response to extracellular ATP, leading to the induction of motor neuron death in co-cultures. Furthermore, evidence indicates that endogenous ATP stimulates SOD1^G93A ^astrocytes in basal conditions and contributes to the maintenance of their neurotoxic phenotype.

Non-transgenic astrocytes required multiple stimuli with ATP over several days to induce the neurotoxic phenotype, while a single stimulus with the P2X_7_r-selective agonist BzATP was sufficient to activate astrocytes to induce the same extent of motor neuron death. BzATP is most potent as an agonist for P2X_7_r, but it is also a weaker agonist of P2X_1_r and P2X_3_r [51-53]. The involvement of P2X_7_r was further implicated in the activation of astrocyte neurotoxicity by the antagonist BBG, as it completely inhibited the action of ATP and BzATP. BBG is a selective antagonist for both P2X_7_r and P2X_5_r. [51-53]. Thus, P2X_7_r appears to be the most likely receptor responsible for inducing the neurotoxic phenotype in astrocytes.

We have previously shown that oxidative stress induced by superoxide and nitric oxide forming peroxynitrite in non-transgenic astrocytes leads to a neurotoxic phenotype [19,42]. Here we found that oxidative stress induced by BzATP stimulation mediated the transition of non-transgenic astrocytes to a neurotoxic phenotype, as NOS inhibitors as well as superoxide and peroxynitrite scavengers prevented their neurotoxicity towards motor neurons. In a similar way, Skaper et al showed that P2X_7_r activation in microglia stimulated peroxynitrite production and led to death of co-cultured neurons [54]. Thus, amplification of oxidative stress by P2X_7_r signaling in microglia and astrocytes could lead to the generation of an adverse environment for vulnerable neurons during neurodegenerative processes.

Because SOD1^G93A ^astrocytes in culture display a neurotoxic phenotype that is maintained by chronic oxidative stress and autocrine pro-inflammatory signaling [14,17,19,20,22], we investigated whether they also presented alterations in extracellular ATP signaling. Indeed, our results indicate that SOD1^G93A ^astrocytes display basally augmented extracellular ATP signaling as evidenced by an ATP-dependent neurotoxic phenotype, increased ATP-dependent proliferation, and increased extracellular ATP metabolism. Thus, ATP emerges as an extracellular factor that could chronically maintain the SOD1^G93A ^astrocyte aberrant phenotype in an autocrine/paracrine manner.

We found that SOD1^G93A ^astrocytes degraded ATP faster than non-transgenic astrocytes, ruling out that their basal alteration in ATP signaling could be caused by a decrease in its extracellular degradation, thereby allowing ATP to accumulate near receptors. An increase in ATP degradation could also be induced in non-transgenic astrocytes exposed to BzATP or LPS. We have previously shown that LPS induces a neurotoxic phenotype in astrocytes, leading to motor neuron death [42]. Increased ATP degradation and/or ectonucleotidase upregulation has been previously described in neural tissue after cortical stab wound and acute ischemia [55,56]. This phenomenon might reflect a cellular attempt to prevent over-activation of purinergic receptors during increases in extracellular ATP, thus promoting the return of extracellular ATP signaling to homeostasis.

Degradation of ATP by ectonucleotidases cannot only terminate deleterious ATP signaling, but also initiates ADP and adenosine signaling through P2Y and P1 receptors. To our surprise, in non-transgenic astrocytes, ATP degraded with apyrase, ADP, AMP, or adenosine led to ~35% more motor neuron attachment and survival compared to untreated controls. Because survival is determined 48 hours after plating of the motor neurons freshly isolated from spinal cords, any treatment that increases attachment of motor neurons will result in an increase of motor neuron survival above the untreated control. These results illustrate how the astrocyte phenotype can be modulated from toxic to highly trophic by changing the balance between ATP, ADP and adenosine signaling through P2X, P2Y or adenosine receptors.

In animal models of ALS, proliferative activated astrocytes interact with microglia to accelerate disease progression [57]. Remarkably, we found that modulating ATP signaling in SOD1^G93A ^astrocytes with apyrase or BBG blocked their neurotoxic phenotype, completely preventing astrocyte-mediated death of motor neurons. A role for ATP and P2X_7_r in the SOD1^G93A ^model was recently proposed by D'Ambrosi et al [41], who showed that SOD1^G93A ^microglia are sensitized to BzATP activation. A combination of aberrant ATP signaling in astrocytes and microglia could generate a positive feedback loop driving a sustained inflammatory response in the spinal cord. The results presented here and the findings in SOD1^G93A ^microglia [41] suggest that P2X_7_r inhibition in ALS could slow disease progression by decreasing astrocyte and microglial activation.

Taken together, the present work supports the idea that extracellular ATP acting through P2X_7_r causes astrocytes to develop a neurotoxic phenotype. In SOD1^G93A ^astrocytes evidence suggests that P2X_7_r is basally activated and contribute to their toxicity towards motor neurons. Thus, modulation of astrocyte P2X_7_r during disease could lead to decreased oxidative stress and inflammatory signaling and in turn the switch to a more trophic phenotype towards neurons. A better understanding of ATP and P2X_7_r signaling in astrocytes could contribute to the development of novel protective therapies in ALS and other neurodegenerative diseases where astrocytes are involved.

## Competing interests

The authors declare that they have no competing interests.

## Authors' contributions

MG, PC, LB participated in the design of the study. MG, HP and PC prepared astrocyte and motor neuron cultures and co-cultures. MG collected the co-culture data and carried out all other experiments. All authors reviewed the data and contributed to the preparation of the manuscript. All authors have read and approved the final manuscript.
